# Consumption Habits and Brand Loyalty of Belgian Coffee Consumers

**DOI:** 10.3390/foods11070969

**Published:** 2022-03-27

**Authors:** Zouheir Alsafra, Véronique Renault, Gianni Parisi, Georges Scholl, Bruno De Meulenaer, Gauthier Eppe, Claude Saegerman

**Affiliations:** 1 Mass Spectrometry Laboratory, MolSys Research Unit, University of Liege, Allée de la Chimie 3, B-6c Sart-Tilman, B-4000 Liege, Belgium; zouheir.alsafra@uliege.be (Z.A.); g.scholl@uliege.be (G.S.); g.eppe@uliege.be (G.E.); 2 Research Unit in Epidemiology and Risk Analysis Applied to Veterinary Sciences (UREAR), Fundamental and Applied Research for Animal Health (FARAH) Centre, Faculty of Veterinary Medicine, University of Liege, Boulevard de Colonster 20, B-42 Sart-Tilman, B-4000 Liege, Belgium; vrenault@uliege.be (V.R.); gianni.parisi@uliege.be (G.P.); 3 Department of Food Safety and Food Quality, nutriFOODchem Unit, Faculty of Bioscience Engineering, Ghent University, Coupure Links 653, B-9000 Ghent, Belgium; bruno.demeulenaer@ugent.be

**Keywords:** coffee consumption, brand loyalty, (alkyl)-furans

## Abstract

Coffee is usually subjected to a roasting process which is responsible for the formation of aroma and flavours but also of some undesirable compounds such as furan and alkyl furans. These compounds are known as process contaminants of the roasting process and exhibit some harmful effects. In order to evaluate the exposure to these compounds in coffee, it is necessary to know the levels of contamination as well as consumption habits. The degree of consumers’ loyalty to specific coffee brands could also be an important driver affecting the level of exposure. This research aimed to evaluate the levels of consumption and the degree of loyalty to coffee brands available in Belgian markets, as well as the factors affecting the choice and the consumption of coffee products and coffee brands. Data were collected in Belgium through an online survey. The results show that for the 1930 participants, 87% reported daily coffee consumption and 13% never or occasionally consumed coffee. The global median coffee consumption was 3 cups per day, and the median for individual daily consumers only was 4 cups per day. The level of consumption of ground coffee was about twice higher than coffee beans, followed by instant coffee and relatively very low consumption of coffee substitutes. In total, 78% of participants reported brand loyalty but to different degrees. Two coffee brands sold in Belgian regions were listed together by more than 20% of the survey participants. The most frequent criteria for selecting a specific coffee brand were taste and price, followed by tradition and habit. The age of coffee consumers and several sociodemographic characteristics have significant effects on coffee consumption. The type of coffee product, the degree of loyalty, and also the type of packaging should be further considered (when available) in the exposure assessment to furan compounds.

## 1. Introduction

Coffee is one of the most consumed beverages in the world. Millions of tons of coffee beans are produced annually, making it an important economic source for producing countries [1]. During coffee roasting, complex chemical reactions take place and produce a wide range of chemical compounds considered as a key aroma of coffee [2]. However, some of the compounds formed have potentially harmful effects on human health, and thus, they were classified as processed contaminants such as acrylamide and polycyclic aromatic hydrocarbons (PAHs), as well as the furan and alkyl furans [3] which are addressed in the current work.

Since 1993, furan has been known to be hepatoxic and carcinogenic in rats and mice [4], and in 1995 it was classified by the IARC as a possible human carcinogen [5]. The benchmark dose (BMDL_10_) for neoplastic effects (hepatocellular adenomas and carcinomas) equal to 1.31 mg/(kg_bw_ × day) was derived from a chronic 2-year mouse study [6]. Recently, a toxicological study has suggested that alkyl furans such as 2- and 3-methylfuran also have toxic effects comparable with those of furan due to their structural similarity and thus similar toxicity pathways [7]. Consequently, the evaluation of the human exposure to these compounds in coffee has become an increasing matter of concern.

To evaluate the exposure to a chemical in food, information is needed on the consumption habits of the food as well as on the contamination levels of the chemical [8]. The consumption habits of Belgian coffee consumers are targeted in the current work, while the contamination levels of furan compounds in coffee available in the Belgian market will be evaluated in a further work.

To assess the consumption habits of a product, two main strategies could be carried out: either conducting a field investigation or an online survey. The first one, generally measures the consumption of different foods in detail, but on a limited number of days (usually two days interview), and sometimes in combination with an additional questionnaire such as a food frequency questionnaire [9,10]. Compared with a field investigation, the online survey can also address a wide range of people and focus on the consumption of different food in detail. In addition, it is faster in collecting data and less expensive, but the questions must be targeted in order to obtain a satisfactory response rate [11]. In this paper, the second strategy was used to assess consumption habits of coffee products.

Regarding the contamination levels of furan compounds in coffee products, several scientific papers confirm that they are mainly related to the amount of precursors in green coffee [12] as well as the degree of coffee roasting [13]. In addition, the final amounts of these compounds in a cup of coffee are directly related to the type of coffee [14], as well as to the brewing method [15]. Different types of coffee products are available on the market, such as coffee beans, ground coffee, instant coffee, and coffee substitutes. The highest levels of furan contamination were reported in coffee beans (2410 µg/kg), and ground coffee (1670 µg/kg), while the levels are relatively lower in instant (380 µg/kg) and substitutes (257 µg/kg) [16]. The levels of alkyl furans in coffee are lower than the previous values except for 2-methylfuran, which is usually 3 to 4 times higher than furan [17]. Regarding the brewing methods, the levels of furan compounds in a cup of coffee can be influenced by the type of coffee machine and the packaging of the coffee, where brewing with an automatic machine, for example, can extract furans more efficiently than filter methods [18]. The content with furan for example in a cup of coffee prepared from a fully automatic machine are reported to be within the range 74–99 µg/L, whereas in a cup prepared using filter method they are in the range 47–53 µg/L [18].

Thus, understanding the driving factors influencing the choice of a coffee product can give valuable information for the subsequent assessment of exposure to these contaminants. The current survey investigated coffee drinking habits as well as the type of coffee regularly consumed, the packaging, the reasons for choosing the coffee, and the extent to which consumers were committed to their regular brands.

Beside coffee, smoking is also considered to be another source of exposure to the molecules addressed in this work [19]. Furan concentration can reach 30.3 µg × cigarette^−1^ due to incomplete combustion [20]. Therefore, additional questions were asked in this survey about smoking habits to evaluate any potential effect of smoking on the consumption habits of coffee.

The current study is part of the “MEFURAN” project which aims to assess the exposure of the Belgian population to furan compounds in foods available on the Belgian market. This study had two objectives. First, we aimed to provide a global overview on the habits of coffee consumption in Belgium and also to study the factors that influence its consumption. Secondly, we wanted to evaluate the degree of consumers’ loyalty to a specific coffee brand. The information produced from this study will be used to assess the exposure assessment to furan and alkyl furans in future studies.

## 2. Materials and Methods

### 2.1. Survey Construction

The survey was hosted by the AESA (Association of Epidemiology and Animal Heath) website. It was created using the LimeSurvey online tool and was conducted in three languages: English, French, and Dutch. The survey was pre-tested by a minimum of two persons in each language before final validation. From April 2021, the survey questionnaire was available on the website until the end of May 2021 (Appendix A). The survey links were shared by mail with several Belgian institutes such as the Universities of Liege and Ghent, the Federal Agency for the Safety of the Food Chain (FASFC), and the Federal Public Service Health, Food Chain Safety, and Environment (FPS Health). It was also accessible to all persons residing on Belgian territory via social networks, and it was advertised on the front page of the University of Liège website.

The data were collected and analysed by the Research Unit in Epidemiology and Risk Analysis Applied to Veterinary Sciences (UREAR, FARAH, ULiège).

### 2.2. Measures

The questionnaires asked about coffee drinking habits, the type and the brand of coffee usually consumed by participants and the degree of loyalty to a particular coffee brand, in addition to smoking habits for those who smoke.

#### 2.2.1. Habits of Coffee Consumption

The participants were asked about coffee consumption habits. Three answers were proposed including ‘never’, ‘occasionally’, and ‘daily’. If the answer was ‘daily’, additional questions were asked about the number of cups per day at home and/or at the office.

#### 2.2.2. The Consumption of Different Types of Coffee

Different types of coffee (such as coffee beans, ground, instant, liquid, and coffee substitutes) and different types of packaging (such as coffee bags, pads, capsules, filters, etc.) are available in markets. To evaluate the consumption of these types of coffee, the survey participants were asked about the type of coffee usually consumed at home and/or at work. If the product consumed was ground coffee, another question was asked about the packaging of the ground coffee.

#### 2.2.3. Brand Most Consumed and Loyalty Measures

The term ‘brand loyalty’ may reflect the consumer’s tendency to repeat the purchase of a particular brand. In terms of exposure, loyalty may mean the continuity of the consumer’s exposure to the same amount of contaminants. In this survey, participants were asked whether they used to drink a particular brand of coffee. If the answer was ‘yes’, they were asked about the brand they usually consume and their level of commitment. The response options were ‘not at all’, ‘a little’, ‘somewhat’, ‘a lot’, ‘fully: I don’t drink any other brand’, and ‘I don’t know’.

#### 2.2.4. Reasons of the Brand Selection

Nine factors thought to influence brand choice were investigated in this work. These factors consisted of ‘health benefits’, ‘price’, ‘taste’, ‘packaging’, ‘caffeine level’, ‘organic aspect’, ‘fair trade’, ‘tradition’, and whether it is a ‘local brand’. If an important factor was omitted, an ‘other’ option was possible with the description of the selection criteria to be specified. Participants were asked to rate the importance of each factor by choosing an ordinal value from 0 to 5, where 0: “not considered at all” and 5: “very important”.

#### 2.2.5. Comparison with a Previous National Field Survey

The daily coffee consumption results obtained in this work were compared with those of the Belgian National Food Consumption Survey (BNFCS) database [21]. The BNFCS dataset was provided in the framework of the ‘MEFURAN’ project. In brief, this BNFCS survey was performed in 2014–2015, and information on food consumption was collected from 3200 participants aged 3–64 years and over two non-consecutive interviews. Food classification in the BNFCS dataset was carried out using FoodEx2 codes and ‘GloboDiet’ Food numbers [9] using the international standardized 24 h dietary recall methodology [22].

For the processing of the BNFCS dataset, the following steps were performed: first, the ‘GloboDiet’ food numbers corresponding to coffee and coffee substitutes were used to select the participants who consumed coffee. The frequency of appearance of consumers in each day of the two interview days was then used as an indicator of coffee consumption per interview day. Then, the median of participants’ daily consumption and the global daily consumption were measured.

#### 2.2.6. Other Measures

Others sociodemographic characteristics were investigated such as the age of the participants, gender, type of activity (e.g., studying, working), and the institute to which participants belonged. Participants were classified in four age groups: 18–24, 25–39, 40–54, and ≥55 years old. Smoking behaviours were also assessed using the following variables: smoking habits and the number of cigarettes smoked per day. Responses to these questions were used to evaluate their possible influences on coffee consumption habits, commitment level, and/or coffee brand selection criteria.

### 2.3. Statistical Tests

The effects of variables with a potential effect on coffee consumption habits were assessed using Pearson’s chi-squared test, correspondence analysis (CA), and the Kruskal–Wallis test. The first test was used to compare consumption habits of categorical variables such as age groups [23]. The CA was used to identify associations between levels of different categorical variables, such as the associations between the levels of consumption habits and age groups. The Kruskal–Wallis test was used to compare variables with ordinal values, such as the comparison between reasons for choosing coffee brands.

Poisson regression models were run in Stata SE 14.1^®^ (StataCorp LP, College Station, TX, USA) to evaluate the effect of the selected explanatory variables on the number of cups of coffee consumed per person (count variable). First, due to extra-binomial variability, a univariate analysis was conducted using a negative binomial regression for each variable (gender, type of activity, and age category). Then, a multivariate negative binomial regression analysis was performed using the variables with a *p*-value < 0.10 in the univariate analysis (in order to be conservative) [24]. The model was progressively simplified by removing the least significant variable with a *p* > 0.05. The model was considered complete, either when all variables had a significant *p*-value (< 0.05), or when it could not be further simplified without having a significant difference between the most complex and the simpler model (likelihood ratio test with a *p*-value < 0.05) [24]. The interaction between the variables was also tested. Due to the lack of interaction, the reduced model with no interaction was retained [23].

All analyses were performed using JMP v15.0 (SAS, Cary, NC, USA) and in Stata SE 14.1^®^ (StataCorp LP, College Station, TX, USA) with a significance level of 0.05.

In all measurements, participants who refused answering a question were excluded from the analysis.

## 3. Results

This section describes, discusses, and interprets the results of the survey, consumption habits of coffee and factors influencing these habits, consumption of different types and brands of coffee, the degree of loyalty to the usual brand, and the reasons for brand choice.

### 3.1. Composition of Participant’ Population

In total, 1930 participants responded to the survey, including members of universities (University A—South of Belgium: 32%, University B—North of Belgium: 18%), national scientific institutes’ members (Institute A: 10%, Institute B: 8%), and other unidentified participants living in Belgium (Others: 32%).

In this survey, 40.7% of the participants were men, 59.0% of them were women, and 0.3% were other. The last gender group (*n*: 7) was excluded from some statistical analyses due to its extremely low contribution.

The ages of the participants were mainly between 25 and 54 years old (69% of the population), as shown in Figure S1. Concerning smoking behaviour, 12% of the participants were smokers, 78% non-smokers, while the remaining 10% did not answer the smoking questions.

### 3.2. Coffee Consumption Habits and the Factors Influencing Them

According to the obtained responses, 258 participants never or occasionally drink coffee, which represents about 13% of the sample (Table S1), while the remaining 87% drink coffee on a daily basis.

Factors with potential effects on consumption habits were investigated in this section. These factors include participants’ age groups, gender, smoking habits, type of activity (working or studying), and the institution of origin.

Concerning the age of participants, the results of Pearson chi-squared test of independence performed on Table S1 suggest that coffee consumption habits are different across age groups (ꭓ^2^: 54.88; degrees of freedom [df]: 6; *p*-value < 0.001). The association between consumption habits and consumer’s age groups was assessed using the correspondence analysis (CA) shown in the Figure S2. The first and the second dimension of the CA explain 87% and 13% of the variability in results, respectively. This figure suggests that the probability of occasional coffee consumption habit is higher for younger participants (18–24 years old) than that for older participants.

According to the chi-squared test performed on Table S2, the gender of participants has a significant effect on daily coffee consumption, with higher consumption among men than women (ꭓ^2^: 6.97; df: 2; *p*-value: 0.03).

When analysing the data on smoking habits, it can be seen that the percentage of daily coffee drinkers is slightly higher in the group of smokers (93.3%) than in the group of non-smokers (88.1%) (Table S3). However, a significant dependence was not confirmed based on the chi-squared test (ꭓ^2^: 5.39; df: 2; *p*-value: 0.07).

Regarding the type of activity, the CA highlights that the probability of an occasional consumption habit is greater for ‘students’ than for ‘workers or retired’ (*p*-value < 0.05) (data not shown).

Finally, a significant dependence of coffee consumption with the participant’s institute is shown in Table S4, with the higher daily consumption observed in the ‘Other’ group, with 91.5% of participants drinking coffee daily (ꭓ^2^: 82.76; df: 8; *p*-value < 0.001).

### 3.3. Number of Cups of Coffee Consumed per Day

The global median of coffee consumption of occasional and daily consumers is 3 cups per day, whereas the median of consumption of daily consumers only (*n*: 1672) is 4 cups per day. Compared with these values, the median of the global coffee consumption obtained by the BNFCS achieved in 2014 was 2 cups per day.

**Figure 1:**
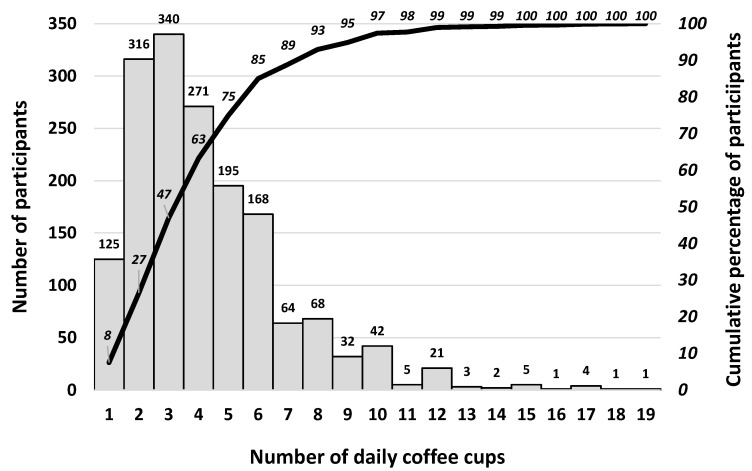
The histogram of coffee consumption of the daily coffee consumers (*n*: 1672).

The distribution of daily coffee consumers is presented in a Pareto chart (Figure 1). This figure shows that 63% of daily coffee drinkers consume up to 4 cups per day.

The multifactorial analysis described in the statistical tests Section 2.3. was performed to evaluate the variables affecting the number of cups of coffee per day consumed by occasional and daily consumers and the interaction between them. The initial model was created using three variables: gender, age category, and type of activity (*n*: 1238). This model displays that type of activity (working/studying status) had no significant effect on coffee consumption with a *p*-value = 0.74. Therefore, a simplified model was created with only the significant variables (gender and age categories). This model highlights the absence of the interaction between these variables. The medians of consumption for age groups are shown in Figure S3. In this figure, the medians of occasional and daily consumption were 2, 3, 4, and 4 cups per day for participants in the groups 18–24, 25–39, 40–54, and ≥55 years old, respectively. For men and women, the medians of occasional and daily consumption were 4 and 3 cups per day, respectively (Figure S4).

### 3.4. Consumption of Different Types of Coffee

The participants were asked whether they usually consumed a particular type of coffee (e.g., beans, ground, instant, coffee substitutes, or liquid coffee (usually cold coffee)). The results reveal that 89% of participants who answered the question consumed a particular type of coffee at home, while this percentage decreased to 55% at work (data not shown).

The distribution of consumption of the different types of coffee as a function of the place of consumption was not homogenous and is displayed in Table 1 (ꭓ^2^: 21.75; df: 3; *p*-value < 0.001).

Regarding the type of packaging, only the ground coffee was addressed in this study, as it is usually sold in different types of packaging such as bags, capsules, pads, and filters.

The distribution of ground consumption based on the choice of packaging is indicated in the Table 2. As shown in this table, the most selected packaging is bags, followed by capsules, pads, and filters. Ground coffee packaging choices are not the same as a function of the place of consumption (ꭓ^2^: 18.48; df: 4; *p*-value = 0.001).

### 3.5. Coffee Brands Most Consumed

Participants were asked two questions about the consumption of a particular coffee brand at home and at work. According to the results, 63% of those who answered the first question (*n*: 1749) consume particular brands at home, while 33% of those who answered the second question (*n*: 1745) consume particular brands at work.

Among the coffee brands mentioned in the responses, two brands were listed by more than 20% of the participants. The first brand is an exported brand and was reported by 16% and 13% consumers at work and at home, respectively, while the second is a local brand consumed by 12% and 8% at work and at home, respectively.

### 3.6. Degree of Loyalty to Usual Brand

To evaluate the degree of loyalty to a usual coffee brand, participants were asked the extent to which they are committed to their regular coffee brands.

The results reveal that 78% of participants who answered the question (*n*: 1653) expressed their loyalty to a specific coffee brand but to different degrees. Indeed, 26% of the respondents were ‘somewhat’ loyal to brand, 20% ‘a little’, 24% ‘a lot’, and 8% ‘fully’ loyal (do not drink any other brand). The remaining 22% were ‘not loyal at all’.

The contributions of men and women in each loyalty category were different and appear in the contingency table presented below (Table S5) (ꭓ^2^: 13.18; df: 4; *p*-value = 0.01). The CA performed to evaluate the association between the genders and loyalty shows that the probability of women being in ‘a little’ or ‘a lot’ loyalty categories is higher than in the other loyalty categories. In contrast, the probability of men being in ‘not at all’ or ‘somewhat’ loyalty categories is higher than that in other loyalty categories (data not shown).

The participants who consumed particular brands were asked to express their degree of loyalty to the brand consumed according to the place of consumption. The degree of loyalty of participants who answered the question is illustrated in the Table 3 and highlights a significant difference between the loyalty to a product consumed at home and at work (ꭓ^2^: 16.22; df: 4; *p*-value = 0.003). The CA shows that the probability of those who consume coffee at home who are in the ‘a lot’ loyalty category is higher than that of those in the other loyalty categories, whereas the probability of those who consume coffee at work who are in ‘not at all’ or ‘a little’ loyalty categories is higher than that of those in the other loyalty categories (data not shown).

### 3.7. Reasons for Choosing Usual Brands

**Figure 2:**
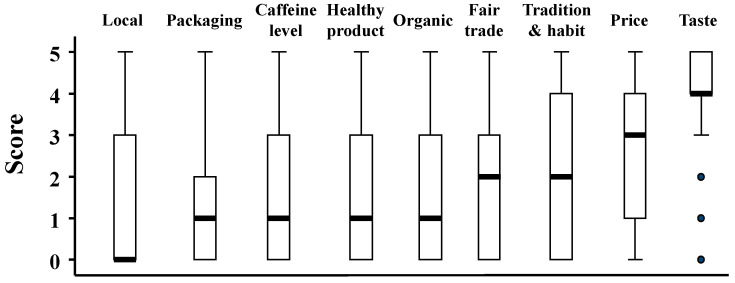
The boxplots for reasons that could influence coffee brand choice (*n*: 1269).

This section investigates in detail the reasons that may influence the decision for selecting a specific coffee brand. Figure 2 shows the boxplots for each reason, with a score between ‘0: not important’ and ‘5: very important’.

As shown in this figure, the ‘taste’ is the main reason for choosing a brand with the highest median score ‘4’, followed by the ‘price’ with a median of ‘3’. Both, the ‘fair trade aspect’ and ‘tradition & habit’ were in third position with a median equal to ‘2’ and non-significant difference between them (Kruskal–Wallis test; *p*-value > 0.05).

Then, ‘caffeine level’, ‘healthy product’, ‘organic’, ‘packaging’ each have a median equal to ‘1’ with no significant difference between them. Finally, the least important reason was whether the coffee is a “local product” or not, with a median equal to ‘0’.

The association between the reasons for brand selection and the gender of participants as well as their ages was also investigated. The results of correspondence analysis (CA) display a significant association between the age and brand choice (ꭓ^2^: 49.19; df: 24; *p*-value = 0.002). The CA displays that the choices of younger participants (18–24 years old) were more related to ‘caffeine level’ than those of older ones, whereas the choices of older participants were more associated with ‘taste’, ‘packaging’, ‘organic’, and ‘fair trade’ aspects (Figure S5).

The CA also displays a significant association between the participants’ gender and reasons for brand selection (ꭓ^2^: 30.72; df: 8; *p*-value = 0.0002). The results of the CA suggest that both men and women select their brands based on ‘taste’, while men are more likely to purchase their brands based on the ‘local’ and ‘fair trade’ aspects than women who select the brands based on the ‘price’ and the ‘organic’ aspects (results not shown).

## 4. Discussion

Based on the obtained results, the habits of coffee consumption are influenced by several factors, such as participants’ gender, age, and place of consumption.

The results obtained in this paper suggest that the consumption of workers is higher than that of students (Section 3.2). However, these results should not be directly associated with the status of worker or student but rather with the age of the participant, as discussed in Section 3.3. A higher median of daily intake was observed for older participants than for younger ones (Figure S3). Based on these results, older consumers should be more exposed to furan compounds in coffee than younger ones.

In addition, although there was no association observed between the gender of participants and their ages, as mentioned in Section 3.3, gender seems to have a significant effect on coffee consumption, with a higher consumption for men than women (Figure S4).

Concerning the place of consumption, the results show that it has a significant effect not only on the habits of coffee consumption (Section 3.4) but also on the choice of coffee product (Table 1). It seems that participants are less free to choose their preferred products at work compared with at home, as shown in Table 3.

Based on this discussion, the exposure to furan compounds in coffee is mainly related to sociodemographic characteristics such as gender, age, and place of consumption. We can also conclude that it is important to take these characteristics into account (when available) for a more realistic risk assessment of furan.

The global median daily coffee intake obtained in the Belgian sample is 3 cups per day, which is slightly higher to the one obtained by the BNFCS conducted in 2014. The difference could be partly explained by the population sampling specific to this work, where the questions were addressed mainly to workers and employees with a higher education level. In addition, the COVID-19 pandemic may also have influenced the coffee intake in 2020. Indeed, the Belgian purchase of coffee beans increased up to 15% per year during the pandemic [25,26]. The exposure to furan compounds in the current pandemic crisis could therefore be higher than in previous years. The number of cups estimated in the current study should be used to evaluate the daily intake of furan compounds in coffee by Belgian coffee consumers.

Although the ‘packaging’ factor did not score highly for brand choice (Figure 2), it could have a significant impact on the choice of the type of coffee. Indeed, ground coffee is usually sold in different types of packaging, such as coffee bags, pads, capsules, and filters, which offers a relatively high variety of ground coffee compared with coffee beans, which are usually sold in coffee bags. This could explain the results shown in Table 1, where the level of consumption of ground coffee is about twice as high as that of coffee beans. An additional factor that may justify this difference is the price of a coffee maker, as the price of a coffee maker and a beans grinder is usually more expensive than a simple drip coffee maker for ground.

The most consumed type of coffee remains ground coffee, followed by coffee beans, instant coffee, and finally, liquid coffee and coffee substitutes. In terms of exposure to furan compounds, the type of packaging should be taken into account when assessing the risk, as the final concentration of these compounds in a cup of coffee depends on the condition of brewing, as mentioned above.

In addition, the distribution of coffee consumers according to the type of coffee and packaging (Table 1 and Table 2) could be useful for creating a representative sampling design and for constructing a dataset on furan contamination, as it reflects the habits of coffee consumers.

It can also be seen that Belgians choose a particular brand of coffee because they like its taste and the pleasure they get from drinking it (Figure 2). Price is also an important factor affecting the brand selection. They also select a particular brand because it is related to family tradition, which is consistent with previous studies [27].

It is also interesting to note that younger participants select their brands based on the level of caffeine to benefit from its stimulating effect, while older participants choose mainly on the basis of ‘taste’, ‘organic’, and ‘fair trade’ aspects (Figure S5).

The degree of loyalty of the studied Belgian sample to specific brands varied from ‘not at all’ to ‘fully loyal’. It can be concluded that the studied sample display slight characteristics of coffee loyalty, with 24% ‘a lot loyal’ and 8% ‘fully loyal’, whereas the remaining participants do not have clear preferences for brands. In term of exposure, the level of exposure to furan compounds in coffee is not directly related to the brand but rather to the conditions of roasting and brewing, as well as to the amount of furans precursors in green coffee, as discussed above. However, the degree of consumer attachment to a particular brand could also determine the systematic exposure to the contaminant, as the green coffee of a particular brand is often provided from the same origin and roasted under similar conditions. Therefore, both the type of coffee and the degree of loyalty (when it is available) should be taken into account in further exposure assessments.

## 5. Conclusions

The results obtained in this study suggest that coffee consumption is influenced by several factors that depend mainly on the age of the consumer, gender, and place of consumption. Some of these factors such as age groups and gender might be easily introduced in a model to achieve an accurate evaluation of furan exposure. The global median of coffee intake of the Belgian participants in this work is 3 cups per day. This value should be considered when performing a realistic evaluation of the exposure of Belgian coffee consumers to furans in coffee. This intake is slightly higher than that obtained in a previous consumption survey, which might be due to the increased demand for coffee products associated with the current COVID-19 pandemic and crisis. The type of coffee product, the degree of loyalty, and the packaging should be recommended to be taken into account when assessing exposure to furan compounds. The distribution of coffee consumers according to coffee type and packaging could be used to allocate coffee samples in a sampling plan created to construct a furan contamination dataset. Taste, price, tradition, and habits are the main driving factors influencing the selection of a particular coffee brand.

## Figures and Tables

**Table 1:** The number of participants consuming different types of coffee at home and at work.

Coffee Type	Place of Consumption	
	Work (*n*: 922)	Home (*n*: 1560)
Ground coffee (ground, capsules, pads, and filters)	530 (57.5%)	1015 (65.1%)
Coffee beans	310 (33.6%)	483 (31.0%)
Instant coffee	67 (7.3%)	57 (3.6%)
Coffee substitutes	3 (0.3%)	5 (0.3%)
Liquid coffee	12 (1.3%)	0

**Table 2:** The number of participants using one or several types of ground coffee packages.

Packages of Ground Coffee	Place of Consumption	
	Work (*n*: 529)	Home (*n*: 1014)
Bags	200 (37.8%)	478 (47.1%)
Capsules	144 (27.2%)	280 (27.6%)
Pads	133 (25.1%)	186 (18.3%)
Filters	43 (8.1%)	57 (5.6%)
Several types of packaging	9 (1.7%)	13 (1.3%)

**Table 3:** The number of participants expressing their loyalty to brands offered at home or at work.

Place	Loyalty Categories				
	A Little	A Lot	Fully	Not at All	Somewhat
At home *n*: 1152	189 (16%)	379 (33)	125 (11%)	121 (11%)	338 (29%)
At work *n*: 577	103 (18%)	170 (29)	46 (8%)	95 (16%)	163 (28%)

## Data Availability

The data that support the findings of this study are available from the corresponding author upon reasonable request.

## Supplementary Materials

The following supporting information can be downloaded at: https://www.mdpi.com/article/10.3390/foods11070969/s1, Figure S1: The age pyramid of the survey participants (n: 1930), Figure S2: The correspondence analysis for consumption habits and age groups (n: 1930), Figure S3: The box plots of daily cups in different age groups for occasionally and daily coffee consumers (n: 1845), Figure S4: The box plots of occasionally and daily coffee consumption (cups/day) for men and women (n: 1838), Figure S5: The correspondence analysis for brand selection criteria and age groups (n: 1263), Table S1: The number of participants in each age group according to coffee consumption habits (n: 1930), Table S2: The number of men and women according to coffee consumption habits (n: 1923), Table S3: The frequency of coffee consumption for smokers and non-smokers (n: 1721), Table S4: The habits of coffee consumption in different Belgian institutes (n: 1930), Table S5: The number of men and women in each loyalty category (n: 1653).

## Appendix A. Consumption Survey Regarding Coffee and Tobacco Consumption to Determine the Level of Exposure to Furan and Methylfurans

Dear Participant, The objective of the study is to determine the level of exposure to furan and methylfurans through coffee and tobacco consumption. The survey will consist of answering a 10 to 15 min questionnaire related to your coffee and tobacco consumption habits. This study is being carried out by the Universities of Liege and Ghent. By taking part in this study, you acknowledge that you have read and understood the information on the study below. Please ensure you have read and understood this information before continuing. Participation is open to persons aged 18 or older, residing in Belgium, and belonging to the University of Liege or Ghent as staff or student. It is entirely voluntary and anonymous. The data will be collected and analysed by the Unit of Research in Epidemiology and Risk Analysis. Your data might be shared, but they will be completely anonymous, and it will not be possible to identify you individually from your answers. If you are concerned about this study or about the manner your data are processed, or if you wish to contact us about your rights, please first contact Prof. Claude Saegerman at the following address: claude.saegerman@uliege.be		
**Code**	**Question (EN)**	**Attributes**
**Sociodemographic characteristics**		
G1	In which university are you working or studying?	- Liege - Ghent
G2	What is your gender?	Male/Female/Other
G3	How old are you?	- 18 to 24 years - 25–39 years - 40–54 years - 55 years or more
G4	Are you:	- A student - An administrative staff - A technical staff - A scientific and/or academic staff - Other (if so please specify)
**Coffee consumption**		
C1	How frequently do you drink coffee?	- Never - Occasionally - Daily
C2	If ‘daily’: How many cups of coffee do you drink everyday:	
C2a	At home?	
C2b	At the office?	
**Brand loyalty**		
B1	Do you consume usually a particular type of coffee product (e.g., beans, grounded, instantaneous)	
B1a	At home?	Yes/No/I don’t know
B1b	At the office?	Yes/No/I don’t know
	(If yes to B1a), Which type of coffee product do you consume at home (the most frequently)?	- Coffee beans - Ground Coffee - Coffee substitute - Instant - Liquid - Other (if so please define) - I don’t know
	(If yes to B1b), Which type of coffee product do you consume at the office (the most frequently)?	
B2	Do you usually consume a particular brand of coffee?	
B2a	At home?	Yes/No/I don’t know
B2b	At the office?	Yes/No/I don’t know
	(If yes to B2a), What brand of coffee do you consume usually at home?	Exhaustive list to provide as a drop-down menu + ‘I do not know’ and ‘Other’
	(If yes to B2b), What brand of coffee do you consume usually at the office?	
B3	To what extent are you committed to your regular brand of coffee?	- Not at all - A little - Somewhat - A lot - Fully (I do not drink any other brand) - I don’t know - No answer
B4	In choosing your usual brand, what is the importance of each of the following criteria? *Attribute number from 0 to 5 to each criterion with ‘0’ if not considered at all and ‘5’ if the criterion is determinant by itself*	
	Healthy product	Scale from 0 to 5 (+option ‘I do not know’) with ‘0’ if not considered at all and ‘5’ if determinant
	Price	
	Taste	
	Packaging	
	Caffeine level	
	Organic origin	
	Fair trade	
	Tradition/habit	
	Local origin	
	No choice (e.g., at work)	
B4a	Is there any important criterion used to choose your brand which had not been listed in the previous question?	Yes/No
B4b	(If yes to B4a), Please specify what they are	
**Tobacco consumption**		
T1	Do you smoke (daily or occasionally)?	Yes/No
T2	(If yes to T1) How frequently?	- Occasionally (less than one per day) - 1 or 2 per day - 3 to 5 per day - 5 to 10 per day - More than 10 per day
T3	(If yes to T1)What do you smoke?	- Manually rolled cigarettes - Cigarettes with filters - Cigarettes without filters - Cigar or cigarillo - Pipe - Other (precise)
Thank you for your participation in this survey. If you are concerned about this study or about the manner in which your data are processed, or if you wish to contact us about your rights, please first contact Prof. Claude Saegerman at the following address: claude.saegerman@uliege.be		
