# Supplementary material for: Consumption Habits and Brand Loyalty of Belgian Coffee Consumers
Source: Foods. 2022 Mar 27;11(7):969. doi: 10.3390/foods11070969 (PMC8997902; doi:10.3390/foods11070969)
Supplement: Supplementary file 1 [file foods-11-00969-s001.zip › foods-1610202supplementary 3.pdf]

Supplementary Materials

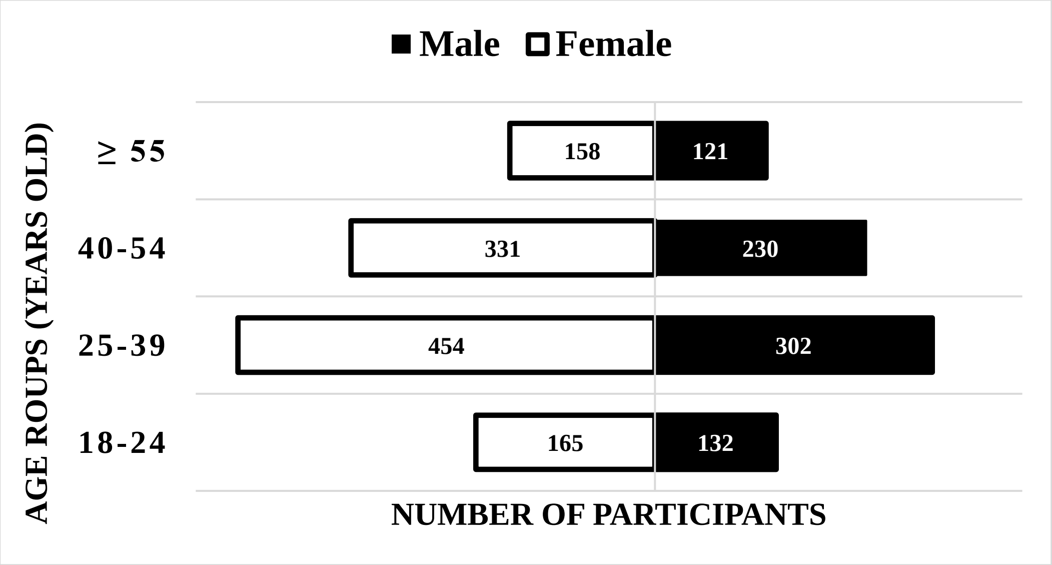

Figure S1: The age pyramid of the survey participants (*n*: 1930)

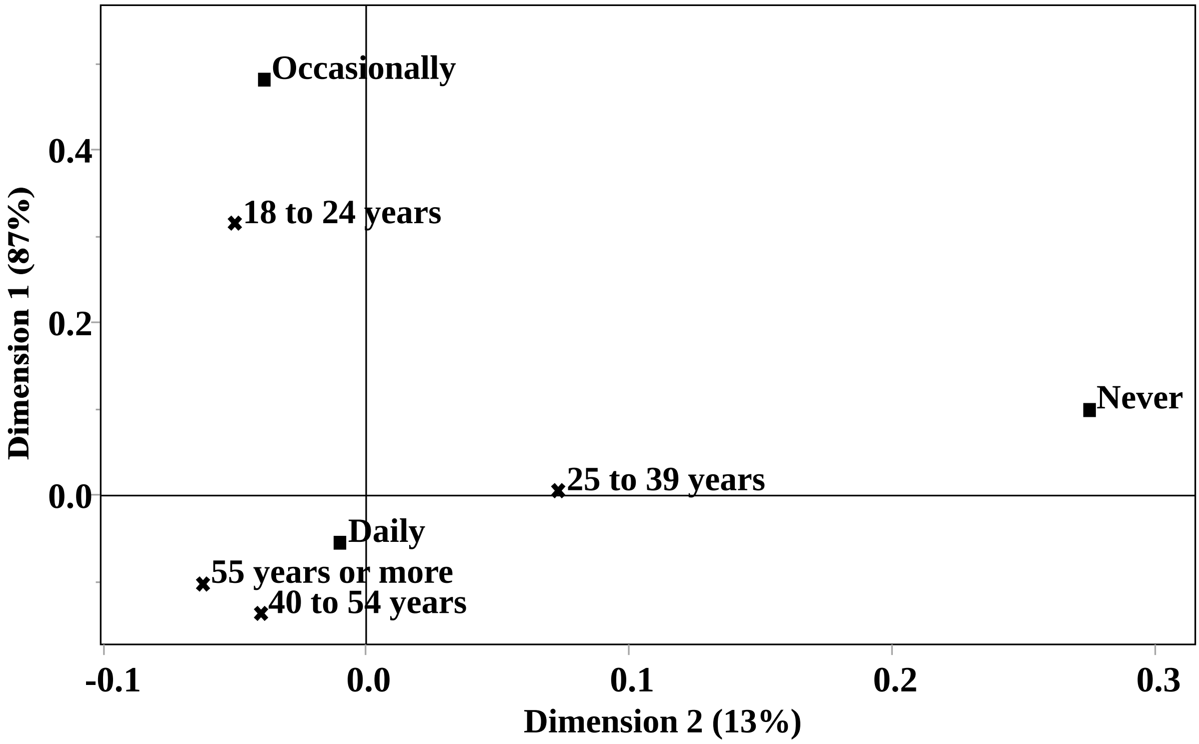

Figure S2: The correspondence analysis for consumption habits and age groups (*n*: 1930)

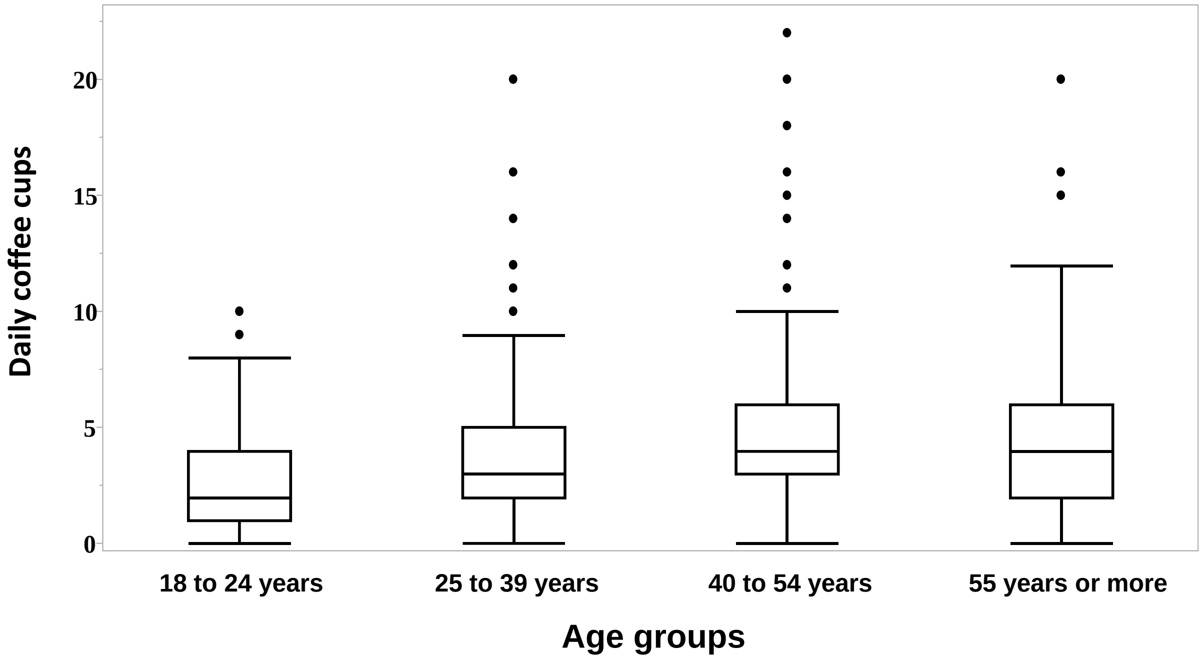

Figure S3: The Box plots of daily cups in different age groups for occasionally and daily coffee consumers (*n*: 1845)

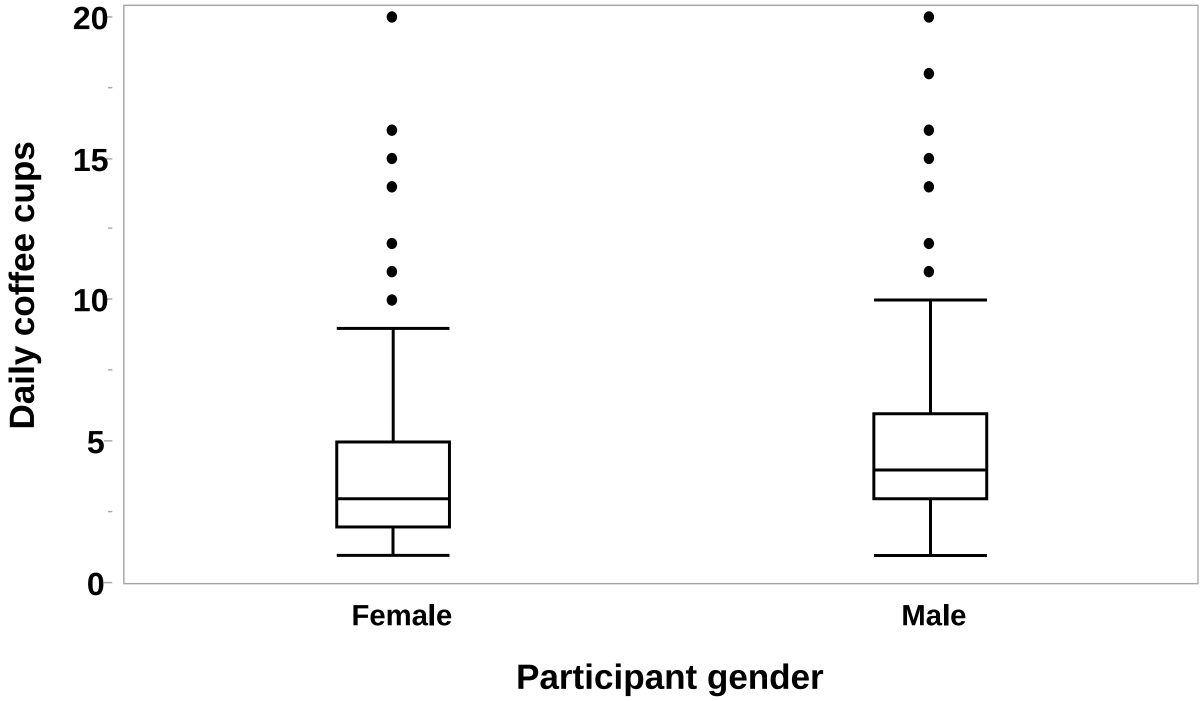

Figure S4: The Box plots of occasionally and daily coffee consumption (cups / day) for men and women (*n*: 1838)

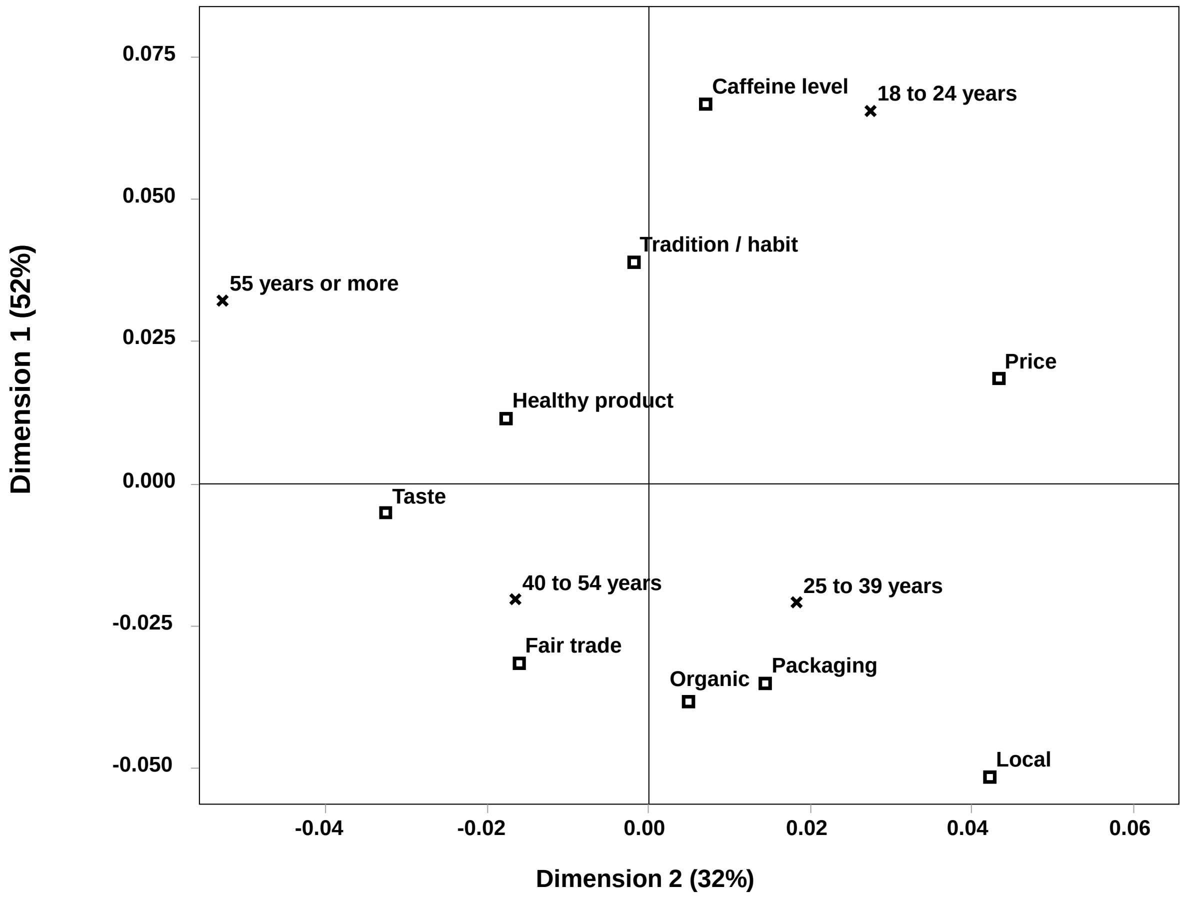

Figure S5: The correspondence analysis for brand selection criteria and age groups (*n*: 1263).

Table S1: The number of participants in each age group according to coffee consumption habits (*n*: 1930)

		Coffee consumption habits		
	**Group**	**Daily (*n*: 1672)**	**Never (*n*:85)**	**Occasionally (*n*: 173)**
**Age group (years old)**	**18-24,** *n*:327	254 (77.7%)	14 (4.3%)	59 (18.0%)
	**25-39,** *n*:762	651 (85.4%)	45 (5.9%)	66 (8.7%)
	**40-54,** *n*:561	513 (91.4%)	18 (3.2%)	30 (5.4%)
	**>55,** *n*:280	254 (90.7%)	8 (2.9%)	18 (6.4%)

Table S2: The number of men and women according to coffee consumption habits (*n*: 1923)

Gender	Daily	Never	Occasionally
**Women,** *n*:1138	975 (85.7%)	62 (5.4%)	101 (8.9%)
**Men,** *n*:785	690 (87.9%)	23 (2.9%)	72 (9.2%)

Table S3: The frequency of coffee consumption for smokers and non-smokers (*n*: 1721)

	**Participants' consumption (%)**		
	**Daily**	**Never**	**Occasionally**
**Non-smokers** *n*:1497	1319 (88.1%)	44 (2.9%)	134 (9.0%)
**Smokers** *n*:224	209 (93.3%)	3 (1.3%)	12 (5.4%)

Table S4: The habits of coffee consumption in different Belgian institutes (*n*: 1930)

		**Participants' consumption (%)**		
	**Group**	**Daily**	**Never**	**Occasionally**
**Institutes**	**Institute (A)** *n*:204	147 (72.1%)	31 (15.2%)	26 (12.7%)
	**Institute (B)** *n*:145	122 (84.1%)	9 (6.2%)	14 (9.7%)
	**University (A)** *n*:618	530 (85.8%)	23 (3.7%)	65 (10.5%)
	**University (B)** *n*:340	303 (89.1%)	11 (3.2%)	26 (7.6%)
	**Other** *n*:623	570 (91.5%)	11 (1.8%)	42 (6.7%)

Table S5: The number of men and women in each loyalty category (*n*: 1653)

		**Loyalty categories**				
		**A little**	**A lot**	**Fully**	**Not at all**	**Somewhat**
**Gender**	**Women**	206 (61.9%)	253 (64.0%)	75 (58.1%)	195 (52.5%)	235 (55.3%)
	**Men**	127 (38.1%)	143 (36.0%)	54 (41.9%)	175 (47.5%)	190 (44.7%)
